# Impact of fremanezumab on disability outcomes in patients with episodic and chronic migraine: a pooled analysis of phase 3 studies

**DOI:** 10.1186/s10194-022-01438-4

**Published:** 2022-08-29

**Authors:** Peter McAllister, Joshua M. Cohen, Verena Ramirez Campos, Xiaoping Ning, Lindsay Janka, Steve Barash

**Affiliations:** 1 New England Institute for Neurology and Headache – Neurology, 30 Buxton Farm Road, Suite 230, Stamford, CT 06905 USA; 2 Teva Branded Pharmaceutical Products R&D, Inc., West Chester, PA USA

**Keywords:** Migraine, Disability, MIDAS, HIT-6, Fremanezumab, Preventive

## Abstract

**Background:**

Migraine is the second leading cause of disability worldwide. Although many preventive treatments reduce migraine frequency and severity, it is unclear whether these treatments reduce migraine-related disability in a clinically meaningful way. This pooled analysis evaluated the ability of fremanezumab to reduce migraine-related disability, based on responses and shifts in severity in patient-reported disability outcomes.

**Methods:**

This pooled analysis included 3 double-blind phase 3 trials (HALO EM, HALO CM, FOCUS) in which patients with episodic or chronic migraine were randomly assigned 1:1:1 to quarterly or monthly fremanezumab or matched placebo for 12 weeks. Migraine-related disability was assessed using the 6-item Headache Impact Test (HIT-6) and Migraine Disability Assessment (MIDAS) questionnaires. A clinically meaningful improvement in disability was defined per American Headache Society guidelines: for HIT-6, a ≥ 5-point reduction; for MIDAS, a ≥ 5-point reduction when baseline score was 11 to 20 or ≥ 30% reduction when baseline score was > 20. Proportions of patients who demonstrated shifts in severity for each outcome were also evaluated.

**Results:**

For patients with baseline MIDAS scores of 11 to 20 (*n* = 234), significantly higher proportions achieved 5-point reductions from baseline in MIDAS scores with fremanezumab (quarterly, 71%; monthly, 70%) compared with placebo (49%; both *P* ≤ 0.01). For patients with baseline MIDAS scores of > 20 (*n* = 1266), proportions achieving ≥30% reduction from baseline in MIDAS scores were also significantly higher with fremanezumab (quarterly, 69%; monthly, 79%) compared with placebo (58%; both *P* < 0.001). For HIT-6 scores, proportions of patients achieving 5-point reductions from baseline were significantly higher with fremanezumab (quarterly, 53%; monthly, 55%) compared with placebo (39%; both *P* < 0.0001). Proportions of patients with shifts of 1 to 3 grades down in MIDAS or HIT-6 disability severity were significantly greater with quarterly and monthly fremanezumab compared with placebo (all *P* < 0.0001).

**Conclusion:**

Fremanezumab demonstrated clinically meaningful improvements in disability severity in this pooled analysis.

**Trial registrations:**

HALO CM, NCT02621931; HALO EM, NCT02629861; FOCUS, NCT03308968.

## Background

Migraine is 1 of the most common disabling neurological diseases worldwide, with an estimated 1-year prevalence of 15% to 18% [1, 2]. Symptoms of migraine may include unilateral throbbing headache, sensitivity to physical activity or visual or auditory stimuli, and nausea [2, 3]. Migraine attacks can be extremely debilitating and may last several days [2–4]. Since 1990, the number of disability-adjusted life-years for those who suffer from migraine has increased by 24.6% among individuals 10 to 25 years of age and 61.2% among those 25 to 49 years of age [1]. As of 2019, migraine was the second leading cause of years lived with disability (YLD) overall worldwide, and the leading cause of YLD among women younger than 50 years [5]. Patients with migraine who have more severe disability may also experience poorer health-related quality of life [6].

Given the high degree of disability associated with migraine, the American Headache Society (AHS) guidelines include severity of disability among the criteria for migraine prevention, and reduction of disability as 1 of the goals of migraine preventive therapy [7]. Although there are numerous medications that have traditionally been used for the preventive treatment of migraine, such as antihypertensives, anticonvulsants, and antidepressants, none of these was developed specifically to prevent migraines [8]. Adherence and persistence to these treatments are generally low, often due to lack of efficacy and/or poor tolerability [9–12].

The calcitonin gene-related peptide (CGRP) pathway has emerged as an effective therapeutic target for both episodic migraine (EM) and chronic migraine (CM), resulting in a shift in the migraine preventive therapeutic landscape [13]. Inhibition of the CGRP pathway has been shown to treat migraine pain [4, 14], and validation of this pathway has led to the development of several monoclonal antibodies that target either the CGRP ligand or receptor for the preventive treatment of migraine [13].

Fremanezumab is a fully humanized monoclonal antibody (IgG isotype 2∆a) that selectively binds the CGRP ligand [15, 16]. The safety, tolerability, and efficacy of fremanezumab was previously demonstrated in the pivotal phase 3 randomized, double-blind, placebo-controlled HALO EM and HALO CM studies in patients with EM and CM, respectively, as well as in the randomized, double-blind, placebo-controlled, phase 3b FOCUS study in patients with EM or CM with inadequate response to 2 to 4 prior migraine preventive medication classes [17–19]. The efficacy and tolerability of fremanezumab has also been demonstrated in a 12-month extension study for the long-term preventive treatment of EM and CM, which included patients who completed the HALO EM or HALO CM study, as well as new patients [20].

Each of these studies demonstrated improvements in disability based on changes from baseline in patient-reported disability outcomes, the 6-item Headache Impact Test (HIT-6), and/or Migraine Disability Assessment (MIDAS) [17–20]. The AHS Consensus Statement guidelines for determining response to CGRP pathway–targeted monoclonal antibodies for migraine includes using the HIT-6 and MIDAS disability questionnaires, which are validated patient-reported measures of headache disability [7]. The following pooled analysis of data from the HALO EM, HALO CM, and FOCUS studies [17–19] evaluated clinically meaningful reductions in disability outcomes (HIT-6 and MIDAS), based on the AHS Consensus Statement–defined criteria, as well as changes in disability severity with fremanezumab treatment [17–19].

## Methods

### Study design and patients

This pooled analysis included patients from 3 randomized, double-blinded, placebo-controlled, clinical trials of similar design (HALO EM [ClinicalTrials.gov Identifier: NCT02629861], HALO CM [ClinicalTrials.gov Identifier: NCT02621931], and FOCUS [ClinicalTrials.gov Identifier: NCT03308968]) [17–19]. All 3 trials included a screening visit and 28-day baseline period before a 12-week, double-blind, placebo-controlled treatment period.

The study protocols and primary results have been previously reported, and the study design and patient selection criteria are summarized briefly here [17–19]. Participants were eligible for all studies if they were 18 to 70 years of age, with a history of migraine based on the International Classification of Headache Disorders 3 (ICHD-3), with onset at or prior to age 50 years and for at least 12 months prior to screening [17–19]. The HALO EM study included patients with EM (≥ 6 and < 15 headache days per month, with ≥ 4 days fulfilling the ICHD-3 beta criteria for migraine) with or without aura, probable migraine, or use of triptans or ergot derivatives [17]. Patients were included in the HALO CM study if they had CM (≥ 15 headache days per month, with ≥ 8 days fulfilling the ICHD-3 beta criteria for migraine, over a 3-month period) [19] with or without aura, probable migraine, or use of triptans or ergot derivatives. The FOCUS study included patients with EM or CM who had experienced 2 to 4 documented inadequate responses (based on a lack of clinically meaningful improvement, poor tolerability, or contraindication) to any of the following pharmacological classes of migraine preventive medications within the last 10 years: β-blockers, anticonvulsants, tricyclic antidepressants, calcium channel blockers, angiotensin II receptor antagonists, onabotulinumtoxinA, or valproic acid [18].

### Ethics approvals and patient consent

The study protocols used for the trials included in this analysis were approved by relevant ethics committees and institutional review boards [17–19]. Additionally, these trials were conducted in accordance with the International Conference for Harmonization Guidelines for Good Clinical Practice, the Declaration of Helsinki, and relevant national and local regulations. Each patient provided written informed consent before any study procedures or assessments were performed [17–19].

### Randomization and treatment procedures

In the HALO EM and HALO CM studies, randomization was stratified by sex, country, and baseline preventive medication use [17, 19]. In the FOCUS study, randomization was stratified by migraine classification (CM or EM), sex, country, and failure to 2 or 3 migraine preventive classes plus valproic acid or valproate [18]. In these studies, the sponsor, investigator, study staff, and participants were blinded to treatment assignment during the treatment period.

Across all 3 studies, patients with EM or CM were randomly assigned 1:1:1 to receive quarterly fremanezumab (months 1/2/3: 675 mg fremanezumab/placebo/placebo), monthly fremanezumab (months 1/2/3: EM: 225 mg fremanezumab/225 mg fremanezumab/225 mg fremanezumab; CM: 675 mg fremanezumab/225 mg fremanezumab/225 mg fremanezumab), or matched monthly placebo by subcutaneous injection during the 12-week treatment period [17–19].

### Outcomes

HIT-6 and MIDAS are validated patient-reported tools that assess the impact of headache on function and measure migraine-related disability, respectively [21, 22]. HIT-6 utilizes a 6-item questionnaire that is scored on a 5-point Likert scale (6 = never, 8 = rarely, 10 = sometimes, 11 = very often, 13 = always) [22]. Scores can range between 36 and 78, with scores of greater numerical value indicating greater impact [22]. Four groups (referred to here as “disability categories”) have been derived to aid in the interpretation of HIT-6 results: scores ≤49 indicate little or no impact; scores ≥50 to ≤55 indicate some impact; scores ≥56 to ≤59 indicate substantial impact; and scores ≥60 indicate severe impact [22]. HIT-6 scores were evaluated in the HALO CM and FOCUS studies [18, 19].

MIDAS utilizes a 5-item questionnaire that is scored by the number of days affected by headache symptoms [23]. Similar to HIT-6, scores with greater numerical value indicate more severe disability [22, 23]. Scores are stratified into disability grades to aid in the interpretation of MIDAS results: scores ≥0 to ≤5 indicate little to no disability; scores ≥6 to ≤10 indicate mild disability; scores ≥11 to ≤20 indicate moderate disability; and scores ≥21 indicate severe disability [23]. MIDAS scores were evaluated in the HALO EM and FOCUS studies [17, 18].

In this pooled analysis, demographic and baseline characteristics were evaluated. Disability responses, based on HIT-6 and MIDAS scores, were assessed at the end of treatment based on the criteria defined in the AHS Consensus Statement (Table 1) [7]. The proportion of patients with a change in HIT-6 disability category or MIDAS disability grade from baseline at the end of treatment was also assessed, along with the overall proportion of patients with a 1-, 2-, or 3-category shift down in disability category or grade from baseline.

**Table 1:** AHS consensus statement–defined clinically meaningful improvements on the HIT-6 and MIDAS [7]

Assessment tool	Meaningful improvement as defined by AHS Consensus Statement
HIT-6	• Reduction from baseline of ≥5 points
MIDAS	• Reduction of ≥5 points when baseline score is 11–20 (moderate disability) or • Reduction of ≥30% when baseline score is > 20 (severe disability)

*AHS* American Headache Society, *HIT-6* 6-Item Headache Impact Test, *MIDAS* Migraine Disability Assessment

### Statistical analyses

For the assessment of baseline and demographic characteristics, patients included in this pooled analysis were from the safety analysis sets from the HALO EM, HALO CM, and FOCUS studies. The safety populations for all 3 studies included all randomly assigned patients who received ≥1 dose of study drug. For the analyses of disability outcomes, patients included in this pooled analysis were from the HALO EM and HALO CM full analysis set (FAS) and the FOCUS modified intent-to-treat (mITT) populations. The FAS populations for the HALO EM and HALO CM studies and the mITT population for the FOCUS study included all randomly assigned patients who received ≥1 dose of study drug and had ≥10 days of postbaseline efficacy assessments for the primary efficacy endpoint (HALO CM, change from baseline in the monthly average number of headache days of at least moderate severity; HALO EM and FOCUS, change from baseline in the monthly average number of migraine days) [17–19].

For the analyses of HIT-6 score responses and changes in disability severity category, data were pooled from the HALO CM and FOCUS studies. For the analyses of MIDAS scores responses and changes in disability severity grade, data were pooled from the HALO EM and FOCUS studies. HIT-6 and MIDAS score responses were also evaluated for patients with CM (HALO CM population pooled with the population of patients with CM from FOCUS) and patients with EM (HALO EM population pooled with the population of patients with EM from FOCUS) separately. Baseline and demographic characteristics were evaluated separately for the overall populations used for HIT-6 analyses (HALO CM and FOCUS) and for MIDAS analyses (HALO EM and FOCUS studies).

For assessments of baseline and demographic characteristics, continuous variables were summarized using descriptive statistics (mean and standard deviation [SD]) and categorical variables were summarized using counts and percentages. Proportions of patients achieving HIT-6 or MIDAS disability responses per the AHS Consensus Statement, as well as proportions of patients with a shift in HIT-6 or MIDAS disability severity, were summarized using counts and percentages. For all assessments, *P* values for between-group comparisons were based on a Cochran-Mantel-Haenszel (CMH) test stratified by study.

## Results

### Patients and baseline disability severity

**Fig. 1:**
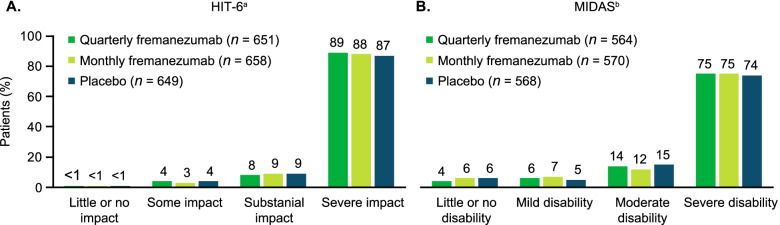
Severity of **A)** headache impact (HIT-6)^a^ and **B)** migraine-related disability (MIDAS)^b^ at study baseline. HIT-6, 6-item Headache Impact Test; MIDAS, Migraine Disability Assessment. ^a^HIT-6 score categories: ≤49 = little or no impact; 50–55 = some impact; 56–59 = substantial impact; 60–78 = severe impact. ^b^MIDAS score grades: 0–5 = minimal or infrequent disability; 6–10 = mild or infrequent disability; 11–20 = moderate disability; ≥21 = severe disability

A total of 1958 patients (placebo, *n* = 649; quarterly fremanezumab, *n* = 651; monthly fremanezumab, *n* = 658) were included in analyses of HIT-6 scores, and 1702 patients (placebo, *n* = 568; quarterly fremanezumab, *n* = 564; monthly fremanezumab, *n* = 570) were included in analyses of MIDAS scores. Across all treatment groups in the population analyzed for HIT-6, the mean age was approximately 43 to 44 years, the majority of patients (86% in all groups) were female, most patients (83–84%) had chronic migraine, and the mean HIT-6 scores was approximately 64 (Table 2). Across all treatment groups in the population analyzed for MIDAS scores, the mean age was approximately 43 to 45 years, the majority of patients (84–85%) were female, most patients had EM (70–71%), and the mean MIDAS score ranged from approximately 49 to 51 (Table 2). Of the patients included in the analyses of MIDAS scores, 234 had moderate disability (MIDAS score, 11–20) at baseline and 1266 had severe disability (MIDAS score, > 20) at baseline. At study baseline, most patients were categorized as experiencing severe impact due to headache and severe migraine-related disability, with ≥80% of patients reporting severe impact on the HIT-6 and ≥ 70% of patients reporting severe disability on the MIDAS (Fig. 1).

**Table 2:** Baseline and demographic characteristics

	HIT-6 analysis population			MIDAS analysis population		
Characteristic	Quarterly fremanezumab (*n* = 652)	Monthly fremanezumab (*n* = 664)	Placebo (*n* = 652)	Quarterly fremanezumab (*n* = 567)	Monthly fremanezumab (*n* = 575)	Placebo (*n* = 570)
Age, years, mean (SD)	43.6 (12.0)	42.9 (11.9)	43.7 (12.0)	43.4 (11.4)	44.5 (11.9)	44.0 (11.9)
Age category, n (%)						
18–45 years	343 (53)	376 (57)	350 (54)	303 (53)	290 (50)	305 (54)
46–65 years	293 (45)	274 (41)	290 (44)	254 (45)	272 (47)	248 (44)
> 65 years	16 (2)	14 (2)	12 (2)	10 (2)	13 (2)	17 (3)
Sex, n (%)						
Female	560 (86)	570 (86)	561 (86)	480 (85)	483 (84)	478 (84)
Male	92 (14)	94 (14)	91 (14)	87 (15)	92 (16)	26.3 (4.7)
BMI, kg/m^2^, mean (SD)	26.0 (4.9)	26.0 (4.8)	26.0 (4.7)	26.0 (4.7)	25.7 (4.8)	
Migraine classification, n (%)						
CM	545 (84)	553 (83)	541 (83)	169 (30)	174 (30)	166 (29)
EM	107 (16)	111 (17)	111 (17)	398 (70)	401 (70)	404 (71)
Baseline HIT-6 score, mean (SD)	64.3 (4.6)	64.3 (4.5)	64.1 (4.9)	–	–	–
Baseline MIDAS score, mean (SD)	–	–	–	51.4 (42.8)	50.0 (44.7)	49.2 (46.2)

*SD* standard deviation, *BMI* body mass index, *CM* chronic migraine, *EM* episodic migraine, *HIT-6* 6-Item Headache Impact Test, *MIDAS* Migraine Disability Assessment

### HIT-6 and MIDAS disability response

**Fig. 2:**
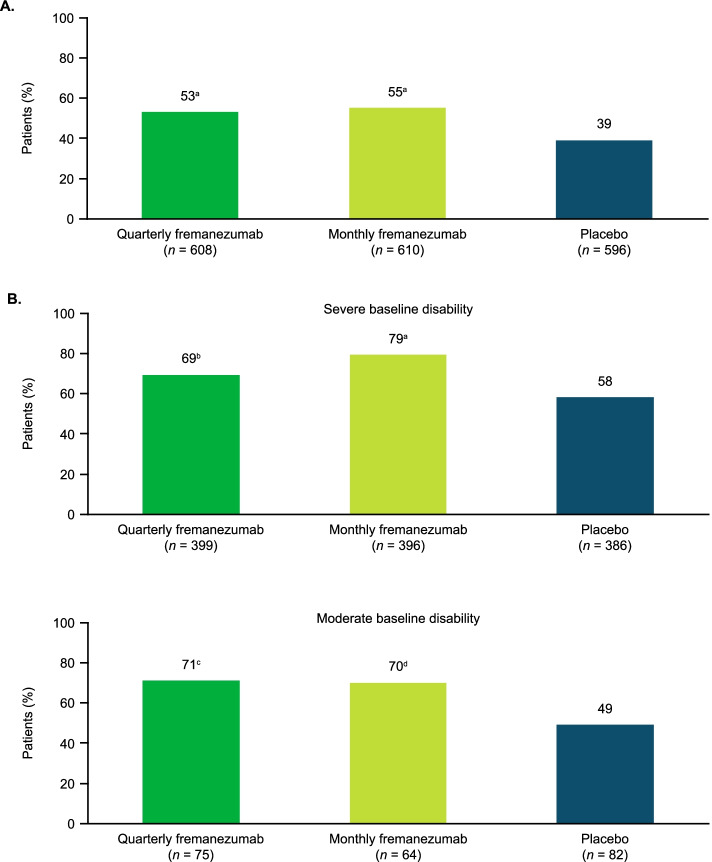
Proportion of patients experiencing **A**) clinically meaningful (≥5-point) reduction in HIT-6 and **B**) clinically meaningful reductions in MIDAS scores during 12 weeks of treatment. HIT-6, 6-item Headache Impact Test; MIDAS, Migraine Disability Assessment. MIDAS: severe baseline disability = baseline MIDAS score, > 20; moderate baseline disability = baseline MIDAS score, 11–20; clinically meaningful reduction in MIDAS score = ≥30% reduction for severe disability and ≥ 5-point reduction for moderate disability. n values shown are the number of patients with data available for analysis of change in HIT-6 or MIDAS scores at the end of treatment. ^a^*P* < 0.0001 versus placebo. ^b^*P* = 0.0006 versus placebo. ^c^*P* = 0.0093 versus placebo. ^d^*P* = 0.0137 versus placebo

The proportion of patients with a clinically meaningful reduction from baseline during 12 weeks of double-blind treatment in the HIT-6 score, per AHS Consensus Statement criteria (≥5-point reduction), was significantly higher with both quarterly fremanezumab (53%) and monthly fremanezumab (55%) compared with placebo (39%; *P* < 0.0001 for both comparisons; Fig. 2A).

Among patients with moderate disability at baseline (MIDAS score, 11–20) and among those with severe disability (MIDAS score, > 20), the proportion of patients with a clinically meaningful reduction from baseline during 12 weeks of double-blind treatment in the MIDAS score, per AHS Consensus Statement criteria (moderate disability, ≥5-point reduction; severe disability, ≥30% reduction), was significantly higher with both fremanezumab dosing regimens compared with placebo (*P* < 0.05 for all comparisons; Fig. 2B). For patients with severe disability at baseline, 69% and 79% experienced a ≥ 30% reduction from baseline in the MIDAS disability score during 12 weeks of double-blind treatment in the quarterly and monthly fremanezumab groups, respectively, compared with 58% in the placebo group. For patients with moderate disability at baseline, 71% of patients in the quarterly fremanezumab group and 70% of patients in the monthly fremanezumab group reported a ≥ 5-point reduction from baseline in the MIDAS disability score during 12 weeks of double-blind treatment compared with 49% in the placebo group.

For patients with CM (*n* = 1630), the proportion of patients with a clinically meaningful (≥5-point) reduction from baseline during 12 weeks of double-blind treatment in the HIT-6 score was significantly higher in the quarterly fremanezumab group (51%) and the monthly fremanezumab group (53%) compared with the placebo group (39%; *P* ≤ 0.0001 for both comparisons). For patients with EM and moderate disability (MIDAS score, 11–20) at baseline (*n* = 191), the proportion of patients with a clinically meaningful (≥5-point) reduction from baseline during 12 weeks of double-blind treatment was significantly higher with quarterly fremanezumab (76%) and monthly fremanezumab (74%) compared with placebo (52%; *P* = 0.0068 and *P* = 0.0275, respectively). For patients with EM and severe disability (MIDAS score, > 20) at baseline (*n* = 842), the proportion of patients with a clinically meaningful (≥30%) reduction from baseline during 12 weeks of double-blind treatment was significantly higher with quarterly fremanezumab (80%) and monthly fremanezumab (86%) compared with placebo (68%; *P* = 0.0025 and *P* < 0.0001, respectively).

### HIT-6 and MIDAS disability severity category shifts

**Fig. 3:**
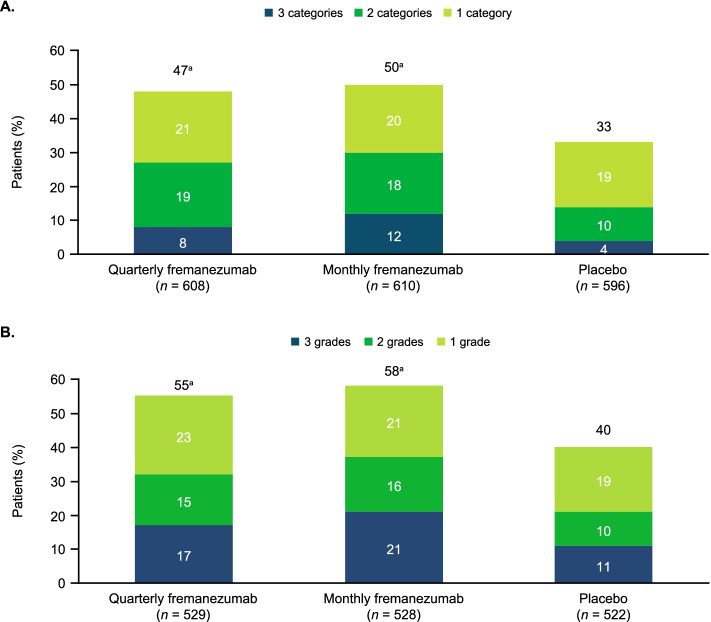
Proportion of patients experiencing downward shifts in **A**) HIT-6 severity categories and **B**) MIDAS severity grades during 12 weeks of treatment. HIT-6, 6-item Headache Impact Test; MIDAS, Migraine Disability Assessment. n values shown are the number of patients with data available for analysis of shift in disability severity category in HIT-6 or MIDAS scores at the end of treatment. Total proportion of patients with a downward shift of 1, 2, or 3 categories or grades (as shown at the top of each bar) may differ from the sum of the proportions of patients with downward shifts of 1, 2, or 3 categories or grades due to rounding. ^a^*P* < 0.0001 versus placebo

The proportion of patients who experienced a downward shift of 1, 2, or 3 severity categories in HIT-6 scores from baseline during 12 weeks of double-blind treatment (indicating a decrease in impact/disability) was significantly greater with both dosing regimens of fremanezumab (47–50%) versus placebo (33%; *P* < 0.0001 for both comparisons; Fig. 3A). No change in HIT-6 severity category was observed in 51% and 47% of patients in the quarterly and monthly fremanezumab groups, respectively, and 62% of patients in the placebo group. Very few patients (2–4% across all treatment groups) experienced an increase in HIT-6 severity category.

For MIDAS scores, a downward shift of 1, 2, or 3 grades in disability severity from baseline during 12 weeks of double-blind treatment was observed for a significantly greater proportion of patients in both fremanezumab treatment groups (55–58%) compared with placebo (40%; *P* < 0.0001 for both comparisons; Fig. 3B). In the quarterly fremanezumab, monthly fremanezumab, and placebo groups, respectively, no change in the MIDAS severity grade was observed in 42%, 38%, and 53% of patients. Across all treatment groups, few patients (3–6%) experienced an increase in MIDAS severity grade.

## Discussion

Both EM and CM are associated with considerable disability, which can have a substantial negative impact on quality of life for those affected [24]. The World Health Organization considers a day lived with severe migraine to be as disabling as a day lived with dementia, quadriplegia, or acute psychosis [24]. Therefore, reducing disability associated with migraine is an important goal of any preventive treatment regimen for EM and CM [7].

Fremanezumab has previously demonstrated favorable tolerability and efficacy in patients with EM and CM, including those with difficult-to-treat migraine based on inadequate response to up to 4 prior migraine preventive medication classes, in randomized, double-blind, placebo-controlled clinical trials [17–19]. In the HALO CM and FOCUS studies, significantly greater least-squares mean (LSM) reductions from baseline were observed in HIT-6 scores during the 4 weeks after the last dose of double-blind treatment with fremanezumab compared with placebo (*P* < 0.001 for all differences between quarterly and monthly fremanezumab vs placebo) [18, 19]. Similarly, in the HALO EM and FOCUS studies, significantly greater LSM reductions from baseline were observed in MIDAS scores during the 4 weeks after the last dose of double-blind treatment with fremanezumab compared with placebo (*P* ≤ 0.002 for all differences between quarterly and monthly fremanezumab vs placebo) [17, 18]. Further, during a subsequent long-term extension study, continued reductions in disability, based on MIDAS and HIT-6 scores, were observed over an additional 12 months of fremanezumab treatment [20].

The current pooled analysis assessing clinically meaningful improvements in these patient-reported disability outcomes, as well as shifts in disability severity, supported those previous findings showing reductions in disability with fremanezumab treatment. In this pooled analysis, in which the majority of patients had severe disability based on HIT-6 scores at baseline, a significantly higher proportion of patients in both the quarterly and monthly fremanezumab groups demonstrated clinically meaningful reductions in HIT-6 disability scores compared with placebo. Among both patients with moderate and severe disability at baseline based on MIDAS scores, significantly higher proportions of patients achieved clinically meaningful reductions in MIDAS scores with both fremanezumab dosing regimens compared with placebo. Similar results were observed in the pooled subgroups of patients with CM and EM.

A significantly higher proportion of patients also exhibited a 1-, 2-, or 3-category reduction in HIT-6 disability category or MIDAS disability grade with quarterly fremanezumab and monthly fremanezumab compared to placebo.

The ability of a migraine preventive treatment to improve migraine-related disability has been identified as a goal of migraine preventive treatment [7]. In a randomized study of the CGRP receptor–targeting monoclonal antibody erenumab, after 52 weeks of treatment, patients receiving erenumab 70 mg and 140 mg experienced reductions in migraine disability, measured using the Migraine Physical Function Impact Diary. Patient-reported physical function impact scores improved by 5.4 and 5.7 points, respectively, and everyday impact scores improved by 6.9 and 7.1 points, respectively [25]. These results, along with those of other studies showing improvements in patient-reported disability assessments [26–28], suggest that treatment with CGRP pathway–targeting monoclonal antibodies reduces the burden of disability associated with migraine.

This pooled analysis was subject to certain limitations. The patients included in the 3 studies in this pooled analysis generally had severe disability at baseline and may represent a more severely affected population than the general migraine population. In addition, due to the severity of disability at baseline in this pooled population, the number of patients with moderate disability severity available for analysis was limited. Nevertheless, results showing significant improvements in disability with fremanezumab treatment were generally consistent, regardless of baseline disability severity category.

## Conclusion

In this pooled analysis of data from patients with EM and CM, including those with difficult-to-treat migraine, fremanezumab demonstrated statistically significant and clinically meaningful improvements in headache- and migraine-related disability scores after 12 weeks of treatment. These findings support the overall clinical benefits of fremanezumab for reducing migraine symptoms, improving patient outcomes, and providing for a better quality of life.

## Data Availability

All data for this pooled analysis are presented in the current manuscript.

## Abbreviations

AHS: American Headache Society
CGRP: calcitonin gene-related peptide
CM: chronic migraine
CMH: Cochran-Mantel-Haenszel
EM: episodic migraine
FAS: full analysis set
HIT-6: 6-item Headache Impact Test
ICHD-3: International Classification of Headache Disorders 3
MIDAS: Migraine Disability Assessment
mITT: modified intent-to-treat
YLD: years lived with disability

## References

[1] G.B.D. Diseases Injuries Collaborators (2020). Global burden of 369 diseases and injuries in 204 countries and territories, 1990-2019: a systematic analysis for the global burden of disease study 2019. Lancet.

[2] Goadsby PJ, Holland PR, Martins-Oliveira M, Hoffmann J, Schankin C, Akerman S (2017). Pathophysiology of migraine: a disorder of sensory processing. Physiol Rev.

[3] Headache Classification Committee of the International Headache Society (2013). The international classification of headache disorders, 3rd edition (beta version). Cephalalgia.

[4] Edvinsson L (2018). The CGRP pathway in migraine as a viable target for therapies. Headache.

[5] Steiner TJ, Stovner LJ, Jensen R, Uluduz D, Katsarava Z; Lifting the burden: the global campaign against headache (2020) Migraine remains second among the world's causes of disability, and first among young women: findings from GBD2019. J Headache Pain 21(1):137. 10.1186/s10194-020-01208-0 10.1186/s10194-020-01208-0 PMC7708887 33267788

[6] Lipton RB, Liberman JN, Kolodner KB, Bigal ME, Dowson A, Stewart WF (2003). Migraine headache disability and health-related quality-of-life: a population-based case-control study from England. Cephalalgia.

[7] American Headache Society (2019). The American headache society position statement on integrating new migraine treatments into clinical practice. Headache.

[8] Jackson JL, Cogbill E, Santana-Davila R, Eldredge C, Collier W, Gradall A (2015). A comparative effectiveness meta-analysis of drugs for the prophylaxis of migraine headache. PLoS One.

[9] Hepp Z, Dodick DW, Varon SF, Chia J, Matthew N, Gillard P (2017). Persistence and switching patterns of oral migraine prophylactic medications among patients with chronic migraine: a retrospective claims analysis. Cephalalgia.

[10] Hepp Z, Dodick DW, Varon SF, Gillard P, Hansen RN, Devine EB (2015). Adherence to oral migraine-preventive medications among patients with chronic migraine. Cephalalgia.

[11] Hepp Z, Bloudek LM, Varon SF (2014). Systematic review of migraine prophylaxis adherence and persistence. J Manag Care Pharm.

[12] Kawata AK, Shah N, Poon JL, Shaffer S, Sapra S, Wilcox TK (2021). Understanding the migraine treatment landscape prior to the introduction of calcitonin gene-related peptide inhibitors: results from the assessment of TolerabiliTy and effectiveness in MigrAINe patients using preventive treatment (ATTAIN) study. Headache.

[13] Moriarty M, Mallick-Searle T, Barch CA, Oas K (2019). Monoclonal antibodies to CGRP or its receptor for migraine prevention. J Nurse Pract.

[14] Tepper SJ (2018). History and review of anti-calcitonin gene-related peptide (CGRP) therapies: from translational research to treatment. Headache.

[15] Walter S, Bigal ME (2015). TEV-48125: a review of a monoclonal CGRP antibody in development for the preventive treatment of migraine. Curr Pain Headache Rep.

[16] Bigal ME, Dodick DW, Rapoport AM, Silberstein SD, Ma Y, Yang R (2015). Safety, tolerability, and efficacy of TEV-48125 for preventive treatment of high-frequency episodic migraine: a multicentre, randomised, double-blind, placebo-controlled, phase 2b study. Lancet Neurol.

[17] Dodick DW, Silberstein SD, Bigal ME, Yeung PP, Goadsby PJ, Blankenbiller T (2018). Effect of fremanezumab compared with placebo for prevention of episodic migraine: a randomized clinical trial. JAMA.

[18] Ferrari MD, Diener HC, Ning X, Galic M, Cohen JM, Yang R (2019). Fremanezumab versus placebo for migraine prevention in patients with documented failure to up to four migraine preventive medication classes (FOCUS): a randomised, double-blind, placebo-controlled, phase 3b trial. Lancet.

[19] Silberstein SD, Dodick DW, Bigal ME, Yeung PP, Goadsby PJ, Blankenbiller T (2017). Fremanezumab for the preventive treatment of chronic migraine. N Engl J Med.

[20] Goadsby PJ, Silberstein SD, Yeung PP, Cohen JM, Ning X, Yang R (2020). Long-term safety, tolerability, and efficacy of fremanezumab in migraine: a randomized study. Neurology.

[21] Lipton RB, Stewart WF, Sawyer J, Edmeads JG (2001). Clinical utility of an instrument assessing migraine disability: the migraine disability assessment (MIDAS) questionnaire. Headache.

[22] Rendas-Baum R, Yang M, Varon SF, Bloudek LM, DeGryse RE, Kosinski M (2014). Validation of the headache impact test (HIT-6) in patients with chronic migraine. Health Qual Life Outcomes.

[23] Stewart WF, Lipton RB, Dowson AJ, Sawyer J (2001). Development and testing of the migraine disability assessment (MIDAS) questionnaire to assess headache-related disability. Neurology.

[24] Blumenfeld AM, Varon SF, Wilcox TK, Buse DC, Kawata AK, Manack A (2011). Disability, HRQoL and resource use among chronic and episodic migraineurs: results from the international burden of migraine study (IBMS). Cephalalgia.

[25] Goadsby PJ, Reuter U, Hallstrom Y, Broessner G, Bonner JH, Zhang F (2020). One-year sustained efficacy of erenumab in episodic migraine: results of the STRIVE study. Neurology.

[26] Camporeale A, Kudrow D, Sides R, Wang S, Van Dycke A, Selzler KJ (2018). A phase 3, long-term, open-label safety study of Galcanezumab in patients with migraine. BMC Neurol.

[27] Stauffer VL, Dodick DW, Zhang Q, Carter JN, Ailani J, Conley RR (2018). Evaluation of galcanezumab for the prevention of episodic migraine: the EVOLVE-1 randomized clinical trial. JAMA Neurol.

[28] Lipton RB, Tepper SJ, Reuter U, Silberstein S, Stewart WF, Nilsen J (2019). Erenumab in chronic migraine: patient-reported outcomes in a randomized double-blind study. Neurology.

